# Pars plana vitrectomy under air infusion versus conventional fluid infusion for dense vitreous hemorrhage: a retrospective comparative study

**DOI:** 10.1186/s12886-026-05137-5

**Published:** 2026-07-22

**Authors:** Ke Xu, Bi Sun, Ying Zhang, Weiliang Fan, Ronghan Wu

**Affiliations:** The National Clinical Research Center for Ocular Diseases, Eye Hospital, Wenzhou Medical University, Wenzhou, 325027 China

**Keywords:** Vitrectomy under air, Vitreous hemorrhage, Pars plana vitrectomy, Air infusion, Vitreoretinal surgery

## Abstract

**Background:**

Vitreous hemorrhage (VH) remains a significant clinical challenge in vitreoretinal surgery. Conventional pars plana vitrectomy (PPV) under balanced salt solution (BSS) infusion is limited by poor intraoperative visualization when hemorrhagic vitreous mixes with the irrigating solution. Vitrectomy under air infusion has been proposed as an alternative approach that may overcome these limitations. This study aimed to compare the surgical efficiency and short-term outcomes of vitrectomy under air infusion versus conventional BSS infusion in the management of dense vitreous hemorrhage.

**Methods:**

This retrospective comparative study included 32 eyes of 32 patients who underwent 23-gauge PPV for vitreous hemorrhage at a single center between August 2024 and August 2025. Sixteen eyes received air infusion (air group) and 16 eyes received conventional BSS infusion (BSS group). All surgeries were performed by the same surgeon. Primary outcome was effective vitrectomy time. Secondary outcomes included best-corrected visual acuity (BCVA) and central macular thickness (CMT) measured by optical coherence tomography (OCT) at 1 month postoperatively, as well as intraoperative and postoperative complications.

**Results:**

Baseline demographics and clinical characteristics were comparable between groups. The mean effective vitrectomy time was shorter in the air group than in the BSS group (4.17 ± 0.97 min vs. 5.02 ± 1.18 min; *p* = 0.033; Cohen’s d = 0.79). At 1-month follow-up, BCVA (0.336 ± 0.24 vs. 0.366 ± 0.36 logMAR; *p* = 0.785) and CMT (298.815 ± 132.45 vs. 265.750 ± 98.91 μm; *p* = 0.430) were comparable between groups. No intraoperative complications occurred in either group. Postoperative complications at 1 month occurred in 3 eyes (18.8%) in the BSS group and 1 eye (6.3%) in the air group; the study was not powered to compare complication rates.

**Conclusions:**

Vitrectomy under air infusion was associated with a shorter effective vitrectomy time than conventional BSS infusion, while achieving comparable short-term visual and anatomical outcomes. The air–vitreous interface may improve intraoperative visualization of hemorrhagic vitreous. As the two techniques produced equivalent short-term outcomes, the potential advantage of air infusion appears to lie in intraoperative efficiency rather than in superior clinical results. Larger prospective studies with longer follow-up are warranted to confirm these findings.

**Supplementary Information:**

The online version contains supplementary material available at https://doi.org/10.1186/s12886-026-05137-5.

## Background

Vitreous hemorrhage (VH) represents a significant clinical complication in ophthalmology, predominantly caused by proliferative diabetic retinopathy (PDR), retinal vein occlusion (RVO), rhegmatogenous retinal detachment (RRD), and ocular trauma. Among these, PDR is the most prevalent cause, where retinal ischemia drives the release of vascular endothelial growth factor (VEGF) and promotes neovascularization [1–4]. These fragile new vessels are prone to rupture, resulting in VH. RVO, including both central and branch forms, can similarly induce retinal ischemia, neovascularization, and hemorrhage, often accompanied by macular edema [5, 6]. RRD may also present with concurrent VH, particularly in patients with pathologic myopia [7].

Pars plana vitrectomy (PPV) is the established surgical approach for managing VH that does not resolve with conservative treatment [1]. Despite substantial technological advances in vitreoretinal instrumentation and surgical techniques over the past decades [8–13], several challenges persist in VH management. Most critically, hemorrhagic vitreous significantly impairs intraoperative visualization. During conventional vitrectomy under balanced salt solution (BSS) infusion, blood mixes with the irrigating fluid to form diffuse opacification, rendering the vitreous-fluid interface indistinct. This obscured interface impedes efficient vitreous engagement by the cutter, leading to substantial wasted aspiration of BSS and necessitating repeated maneuvers. The visualization challenge is particularly pronounced in eyes with active bleeding. Furthermore, incomplete posterior vitreous detachment (PVD) resulting from poor visualization may contribute to postoperative proliferative vitreoretinopathy (PVR), which remains a significant risk factor for surgical failure [14, 15].

To address these limitations, Voleti et al. first described the concept of vitrectomy under air, providing an alternative to conventional BSS infusion [16, 17]. This technique exploits the refractive index differential between air (1.00) and vitreous tissue, as well as the surface tension of air, to enhance surgical visualization and create a more controlled operative environment. Subsequent studies by Frisina et al. and Karacörlu et al. further supported the safety and potential advantages of air infusion in specific vitreoretinal conditions [18, 19]. However, the application of this technique specifically in VH management remains inadequately studied.

This study aimed to retrospectively compare the intraoperative efficiency and short-term postoperative outcomes between vitrectomy under air infusion and conventional BSS infusion in patients with dense vitreous hemorrhage.

## Methods

### Study design and participants

This retrospective comparative study was conducted in accordance with the tenets of the Declaration of Helsinki and adhered to the Strengthening the Reporting of Observational Studies in Epidemiology (STROBE) guidelines. The study protocol was reviewed and approved by the Institutional Review Board of the Eye Hospital of Wenzhou Medical University (approval number: 2026-087-K-074). Due to the retrospective nature of this study, the requirement for written informed consent was waived by the ethics committee.

We reviewed the medical records of 32 consecutive patients (32 eyes) who underwent 23-gauge PPV for dense vitreous hemorrhage at the Eye Hospital of Wenzhou Medical University between August 2024 and August 2025. Patients were divided into two groups according to the infusion medium used during vitreous removal: 16 eyes underwent vitrectomy under air infusion (air group) and 16 eyes underwent conventional vitrectomy under BSS infusion (BSS group). As this was a retrospective study, patients were not randomly assigned; the choice of infusion medium (air or BSS) was made at the operating surgeon’s discretion and was not based on disease severity or other specific patient characteristics. Consequently, the two groups were broadly comparable at baseline with respect to age, sex, and axial length, although the distribution of etiology differed between groups (see Results). The primary etiologies of VH included PDR, RVO, and RRD; in all PDR eyes the retina was attached. Patients who underwent concurrent cataract extraction with intraocular lens implantation, who had silicone oil–filled eyes, or who had pre-existing tractional retinal detachment were excluded.

### Preoperative assessment

All patients underwent comprehensive ophthalmic evaluation preoperatively, including best-corrected visual acuity (BCVA) measurement, slit-lamp biomicroscopy, intraocular pressure (IOP) measurement, B-scan ultrasonography, and optical biometry for axial length (AL). Demographic characteristics, disease duration, medical history, and lens status were documented.

### Surgical technique

All procedures were performed by a single experienced vitreoretinal surgeon (R.W.) using a 23-gauge transconjunctival sutureless three-port vitrectomy system (Constellation Vision System, Alcon Laboratories, Fort Worth, TX, USA), ensuring consistency in surgical outcomes.

**Fig. 1:**
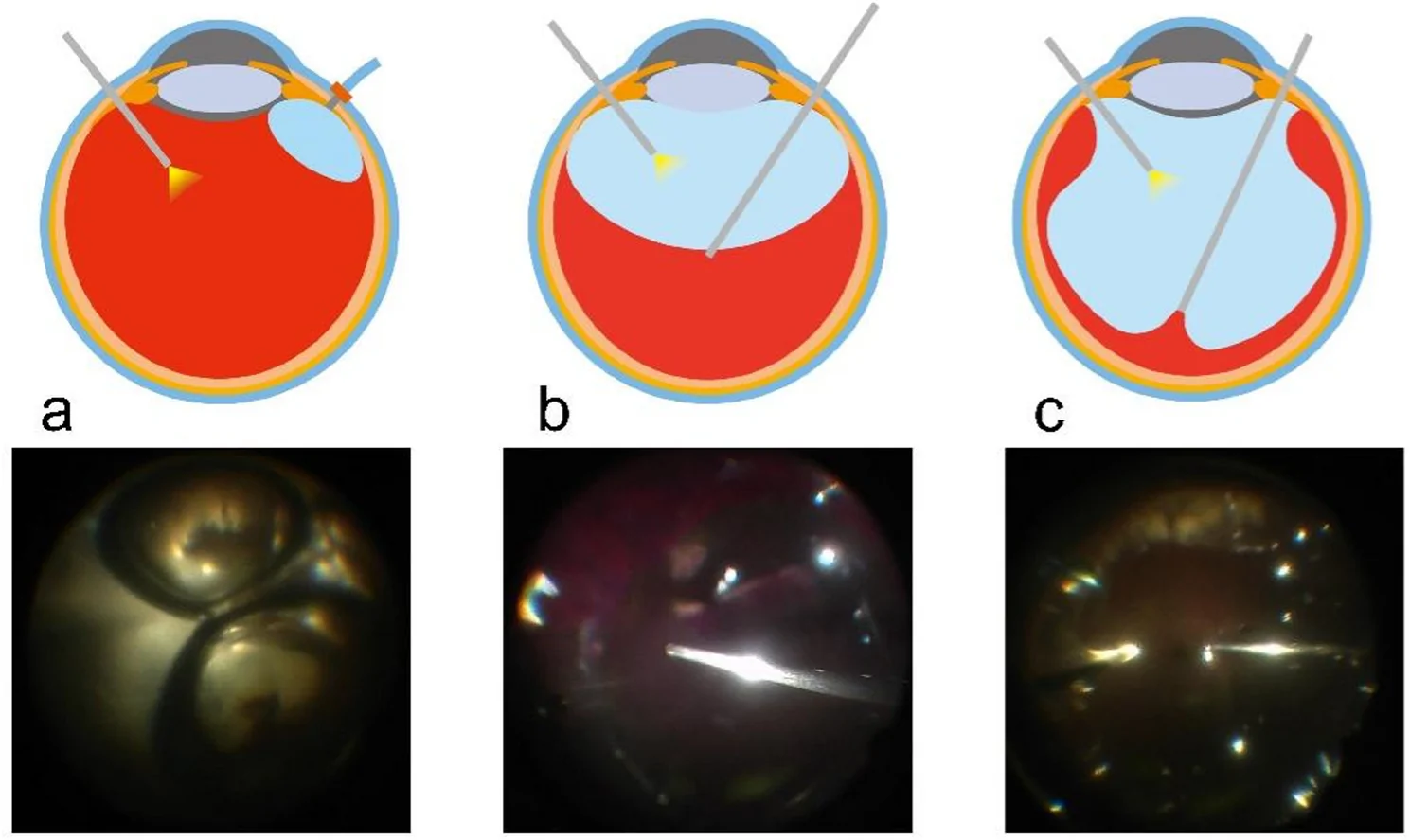
Surgical steps of vitrectomy performed under air infusion. **(a)** Initiation of air infusion. **(b)** Vitreous/air interface establishment. **(c)** Vitreous base excision

#### Air infusion technique (air group)

**Fig. 2:**
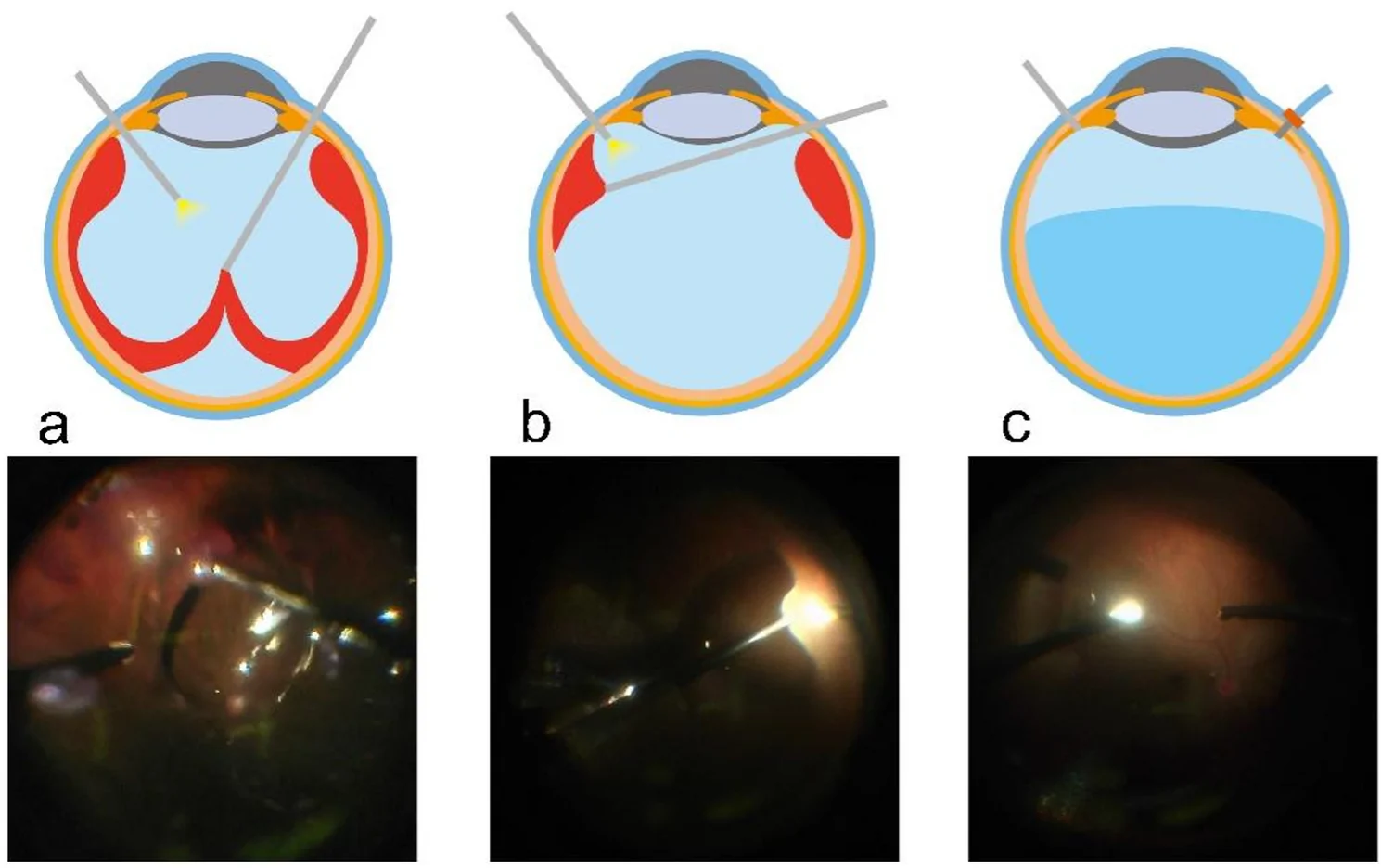
Surgical steps of vitrectomy performed under air infusion. **(a)** Posterior vitreous detachment (PVD). **(b)** Removal of peripheral vitreous. **(c)** Air-BSS fluid exchange

Standard 23-gauge three-port pars plana sclerotomies were performed. Air infusion was initiated at a pressure of 40 mmHg, with a vitrectomy cut rate of 4500 cuts per minute and maximum linear aspiration pressure of 400 mmHg. The air medium facilitated rapid aspiration of hemorrhagic fluid and created a distinct air-vitreous interface, clearly delineating the hyaloid fiber structure and enabling efficient vitreous cortex removal (Fig. 1). Following anterior vitreous excision, air displaced the peripheral and basal vitreous toward the retina. The vitreous cutter was used to aspirate and elevate the posterior vitreous cortex, completing PVD before removing posterior pole vitreous. The expanded field of view under air allowed circumferential peripheral vitrectomy without requiring scleral depression. Once vitreous removal was complete, the air in the vitreous cavity was exchanged for BSS (an air-to-fluid exchange), and all subsequent steps were carried out under BSS infusion as in the BSS group (Fig. 2).

#### Conventional BSS infusion technique (BSS group)

Standard BSS infusion was employed with identical cutting parameters. Dynamic scleral depression assisted in removal of the peripheral vitreous and vitreous base.

In both groups, after core vitreous removal under the respective infusion media, all subsequent steps were performed under BSS infusion. Circumferential internal limiting membrane (ILM) peeling was performed without triamcinolone acetonide assistance. ILM peeling was carried out routinely in all eyes as a standard component of our surgical group’s protocol, with the aim of reducing the risk of postoperative epiretinal membrane formation [20]. Additional procedures including epiretinal membrane removal and panretinal photocoagulation (PRP) were completed under BSS infusion as indicated. If fogging of the posterior lens surface occurred intraoperatively, viscoelastic coating was applied. At the conclusion of surgery, a fluid–air exchange was performed and the vitreous cavity was filled with filtered sterilized air for intraocular tamponade; the same tamponade strategy was used in both groups.

### Outcome measures

The primary outcome was effective vitrectomy time, defined as the net duration of vitreous removal—from the start of infusion (air or BSS) with engagement of the vitreous cutter to the surgeon’s completion of vitreous removal (the last release of the cutter foot pedal at the end of vitrectomy)—excluding the time for dye staining, ILM peeling, endolaser, and membrane removal. Effective vitrectomy time was measured intraoperatively by the surgical assistant using a stopwatch, started when the surgeon engaged the vitreous cutter under infusion and stopped at the surgeon’s final release of the cutter foot pedal upon completion of vitreous removal. Secondary outcomes included: (1) BCVA at 1 month postoperatively, measured in logarithm of the minimum angle of resolution (logMAR); (2) central macular thickness (CMT) measured by spectral-domain OCT at 1 month postoperatively; (3) intraoperative complications; and (4) postoperative complications at 1 month.

### Statistical analysis

Data analysis was performed using SPSS 25.0 (IBM Corp., Armonk, NY, USA). Continuous variables were expressed as mean ± standard deviation and compared between groups using the independent-samples t-test; the effect size for the primary outcome was reported as Cohen’s d. Categorical variables were presented as frequencies and percentages and compared using Fisher’s exact test, which was preferred over the chi-square test because of the small sample size and expected cell counts below five. Continuous variables (effective vitrectomy time, axial length, visual acuity, age, and central macular thickness) were analyzed with the independent-samples t-test, and categorical variables (sex and etiology) with Fisher’s exact test (the Fisher–Freeman–Halton extension for the 3 × 2 etiology table). To address the baseline imbalance in etiology, two additional analyses were performed: (1) a subgroup analysis of effective vitrectomy time excluding RRD eyes—that is, restricted to the vascular, non-detachment etiologies (RVO and PDR), which share a comparable vitreous-hemorrhage state and surgical approach and were therefore analysed together—with a further sensitivity analysis restricted to PDR eyes; and (2) a multivariable linear regression with effective vitrectomy time as the dependent variable and infusion medium and etiology as independent variables. A post-hoc power analysis was performed for the primary outcome. A two-sided P value < 0.05 was considered statistically significant.

## Results

### Baseline characteristics

A total of 32 eyes from 32 patients were included. The BSS group comprised 16 patients (16 eyes) who underwent conventional pars plana vitrectomy under BSS infusion, while the air group included 16 patients (16 eyes) treated with vitrectomy under air. Patient demographics and clinical characteristics are presented in Table 1. The mean age was 61.31 ± 12.86 years in the BSS group and 59.31 ± 9.30 years in the air group (*p* = 0.618). Gender distribution was similar between groups (*p* > 0.9999). Mean preoperative axial length was 23.26 ± 0.89 mm and 23.75 ± 1.34 mm in the BSS and air groups, respectively (*p* = 0.202). Regarding etiology, PDR was the most common diagnosis overall (20 eyes, 62.5%), followed by RVO (8 eyes, 25.0%) and RRD (4 eyes, 12.5%). PDR predominated in the air group (14/16, 87.5%) compared with the BSS group (6/16, 37.5%), whereas all four RRD eyes were in the BSS group. By the Fisher–Freeman–Halton exact test, the distribution of etiology differed significantly between the two groups (*p* = 0.007). Because of this baseline etiologic imbalance, the comparison of effective vitrectomy time was further addressed by a subgroup analysis excluding RRD eyes and by a multivariable regression adjusting for etiology (see below), and the imbalance is considered in the Discussion.

**Table 1:** Demographic data of patients

Characteristics	Overall	Conventional BSS vitrectomy	Vitrectomy under air	Comparison	
	(*n* = 32)	(*n* = 16)	(*n* = 16)	Statistic	*P*
Age(years)				0.504	0.618*
38–50	7(21.9%)	4(25.0%)	3(18.8%)		
50–60	6(18.8%)	2(12.5%)	4(25.0%)		
60–70	12(37.5%)	4(25.0%)	8(50.0%)		
70–82	7(21.9%)	6(37.5%)	1(6.3%)		
Sex				—	> 0.9999^#^
Male	17(53.1%)	9(56.3%)	8(50%)		
Female	15(46.9%)	7(43.8%)	8(50%)		
Etiology				—	0.007^#^
RVO	8(25.0%)	6(37.5%)	2(12.5%)		
PDR	20(62.5%)	6(37.5%)	14(87.5%)		
RRD	4(12.5%)	4(25.0%)	0(0%)		
AL(mm)				1.304	0.202*
23–24	12(37.5%)	6(37.5%)	6(37.5%)		
24–25	14(43.8%)	5(31.3%)	9(56.3%)		
25–26	3(9.4%)	2(12.5%)	1(6.3%)		
26–27	2(6.3%)	2(12.5%)	0(0%)		
> 27	1(3.1%)	1(6.3%)	0(0%)		

independent-samples t-test; ^#^Fisher’s exact test

### Effective vitrectomy time

Effective vitrectomy time was measured for all patients as defined above and compared between the two groups. As shown in Table 2, the mean effective vitrectomy time was 5.02 ± 1.18 min in the BSS group and 4.17 ± 0.97 min in the air group. The air group had a significantly shorter mean effective vitrectomy time than the BSS group (mean difference 0.85 min; *p* = 0.033), corresponding to a medium-to-large effect size (Cohen’s d = 0.79). A post-hoc power analysis for the primary outcome yielded a power of 0.58. No intraoperative complications occurred in either group.

### Subgroup and multivariable analyses

To account for the baseline imbalance in etiology, two additional analyses were performed. First, when the four RRD eyes (all in the BSS group) were excluded and the vascular etiologies (RVO and PDR) were analysed together as a non-detachment subgroup, the mean effective vitrectomy time remained significantly shorter in the air group (4.17 ± 0.97 min, *n* = 16) than in the BSS group (5.24 ± 1.28 min, *n* = 12; mean difference 1.07 min; *p* = 0.018; Cohen’s d = 0.97). In a further sensitivity analysis restricted to PDR eyes (air group, *n* = 14; BSS group, *n* = 6), effective vitrectomy time was shorter in the air group (4.30 ± 0.96 vs. 5.05 ± 1.04 min) in the same direction, although the difference did not reach statistical significance (*p* = 0.135), likely reflecting the small number of PDR eyes in the BSS group (Table 3). Second, in a multivariable linear regression with effective vitrectomy time as the dependent variable, air infusion remained a significant independent predictor of shorter effective vitrectomy time after adjustment for etiology (adjusted β = −1.14 min; 95% CI, − 2.06 to − 0.21; *p* = 0.018), whereas etiology was not a significant predictor (Table 4). Notably, the four RRD eyes in the BSS group did not have longer effective vitrectomy times than the other BSS eyes (mean 4.36 min); their exclusion slightly increased rather than reduced the between-group difference, indicating that the shorter time observed under air infusion is unlikely to be explained by the baseline etiologic imbalance.

### Outcomes

One month postoperatively, BCVA and macular retinal thickness from OCT images were recorded for both groups. As shown in Table 2, the mean postoperative BCVA was 0.336 ± 0.24 logMAR in the air group and 0.366 ± 0.36 logMAR in the BSS group, with no statistically significant difference between groups (*p* = 0.785). The mean central macular thickness was 298.815 ± 132.45 μm in the air group and 265.750 ± 98.91 μm in the BSS group, with no statistically significant difference between groups (*p* = 0.430). The two techniques therefore achieved comparable short-term visual and anatomical outcomes. Postoperative complications at 1 month occurred in 3 eyes (18.8%) in the BSS group (elevated intraocular pressure, *n* = 2; retinal detachment, *n* = 1) and in 1 eye (6.3%) in the air group (retinal detachment, *n* = 1). Macular edema, observed in both groups, was regarded as a manifestation of the underlying retinal vascular disease rather than as a surgical complication and was not counted as such. Because the study was not powered to compare complication rates, this numerical difference (1 vs. 3 eyes) was not formally tested and should be interpreted with caution.

**Table 2:** Intraoperative and postoperative outcomes

Vitrectomy technique	effective vitrectomy time *(Min.)*			BCVA *(1 month postoperative)*			Central macular thickness *(1 month postoperative)*		
	Mean(±SD)	SE(*95%CI*)	*p*	Mean(±SD)	SE(*95%CI*)	*p*	Mean(±SD)	SE(*95%CI*)	*p*
Vitrectomy under air(*n* = 16)	4.17 ± 0.97	0.382 (–1.63 to − 0.07)	0.033	0.336 ± 0.24	0.109 (–0.25 to 0.19)	0.785	298.815 ± 132.45	41.326 (–51.62 to 117.75)	0.430
Conventional BSS vitrectomy(*n* = 16)	5.02 ± 1.18			0.366 ± 0.36			265.750 ± 98.91		

BCVA, Best Corrected Visual Acuity

**Table 3:** Subgroup analyses of effective vitrectomy time

Analysis (set)	Air group, mean ± SD (*n*)	BSS group, mean ± SD (*n*)	Mean difference (min)	*p* value	Cohen’s d
All eyes	4.17 ± 0.97 (16)	5.02 ± 1.18 (16)	0.85	0.033	0.79
Non-RRD (RVO + PDR)	4.17 ± 0.97 (16)	5.24 ± 1.28 (12)	1.07	0.018	0.97
PDR only	4.30 ± 0.96 (14)	5.05 ± 1.04 (6)	0.75	0.135	0.76

Comparisons by independent-samples t-test. RRD eyes (*n* = 4) were all in the BSS group. The non-RRD subgroup combines RVO and PDR eyes (vascular, non-detachment etiologies with comparable surgical handling). Effect size expressed as Cohen’s d

**Table 4:** Multivariable linear regression for effective vitrectomy time

Predictor	β (min)	95% CI	*p* value
Air infusion (vs. BSS)	−1.14	−2.06 to − 0.21	0.018
Etiology: PDR (vs. RVO)	0.17	−0.84 to 1.18	0.736
Etiology: RRD (vs. RVO)	−0.79	−2.16 to 0.58	0.247

Dependent variable: effective vitrectomy time (minutes). Reference categories: BSS infusion and RVO etiology. Model R² = 0.20. β, unstandardized regression coefficient; CI, confidence interval

## Discussion

This retrospective comparative study found that vitrectomy under air infusion was associated with a shorter effective vitrectomy time than conventional BSS infusion in the management of dense vitreous hemorrhage, while the two techniques achieved comparable short-term visual and anatomical outcomes. These findings suggest that air infusion may offer a potential improvement in intraoperative efficiency, although the clinical significance of this time reduction warrants further evaluation. Because the postoperative visual and anatomical results were equivalent between groups, the potential benefit of air infusion appears to relate to intraoperative efficiency and visualization rather than to superior surgical outcomes.

Vitreous hemorrhage is a common pathological condition in ophthalmic practice, typically caused by diabetic retinopathy, retinal vein occlusion, and other retinal vascular diseases. Without timely and effective intervention, these conditions may lead to irreversible visual impairment. Conventionally, PPV has been the primary surgical approach for managing complex vitreous hemorrhage, restoring retinal anatomical structure and function by removing vitreous opacities and associated tractional pathology. However, conventional PPV techniques continue to face considerable challenges regarding intraoperative visualization and retinal protection.

Vitrectomy techniques and instrumentation have advanced tremendously over the past four decades, providing the modern vitreoretinal surgeon with a variety of options in instrument selection [8–10], fluidic systems [11–13], and machine parameters. These advances are designed to achieve the goal of removing as much vitreous as possible in a safe and efficient manner. Optimizing these options is particularly vital for cases requiring complete vitrectomy, such as fibrovascular proliferation and posterior vitreous detachment.

Among the various parameters available, the surgical medium is another adjustable and significant parameter in vitrectomy. Traditionally, most vitrectomy procedures are performed within the vitreous cavity containing a mixture of vitreous and balanced salt solution (BSS). In contrast, an air-filled vitreous cavity is typically reserved for specific maneuvers, such as draining residual subretinal fluid, peripheral endolaser treatment, and retinal tamponade at the conclusion of the surgery. Furthermore, many surgeons prefer to conclude the operation with the vitreous cavity at least partially filled with air to facilitate scleral incision healing, limit the risk of postoperative hypotony, and potentially reduce the risk of infection.

**Fig. 3:**
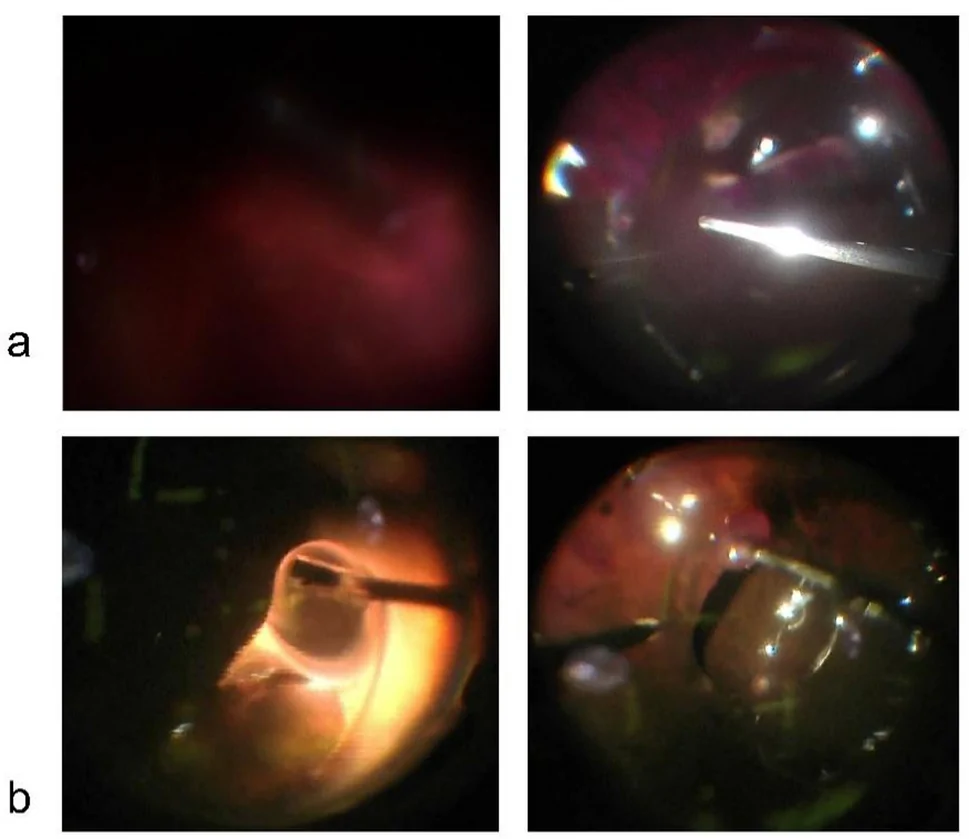
Vitrectomy process in cases of vitreous hemorrhage. **(a)** Left image shows diffuse opacity caused by blood cells dispersed in BSS during conventional vitrectomy; right image shows clear vitreous interface under air infusion. **(b)** Process of vitreous base excision under air infusion, showing distinct vitreous-retinal tissue demarcation during PVD

Therefore, we propose a technique involving premature fluid-air exchange that enables vitrectomy to be performed under air rather than BSS. The significant reduction in effective vitrectomy time (4.17 vs. 5.02 min; *p* = 0.033) observed in the air group can be attributed to several physical properties of the air medium. First, the refractive index differential between air (1.00) and vitreous tissue, compared with the minimal difference between BSS (1.33) and vitreous, creates a distinctly visible air-vitreous interface [16, 17]. In the context of VH, this clearly defined interface facilitates identification and targeted removal of hemorrhagic vitreous, minimizing the ineffective aspiration of irrigating fluid that occurs when surgeons attempt to engage vitreous floating in an aqueous medium (Fig. 3a). During the removal of the vitreous base, the clear vitreous/air interface aids the surgeon in assessing whether any residual posterior vitreous cortex remains, ensuring complete removal (Fig. 3b). More thorough vitreous removal may theoretically reduce the risk of postoperative PVR; however, this hypothesis was not tested in the present study and requires future investigation.

Second, the surface tension of air (approximately 72 mN/m) plays a unique role in VH surgery [16]. This property generates uniform perpendicular pressure across the retinal surface, which helps compress small bleeding points and provides auxiliary hemostatic effects. This is particularly beneficial when managing hemorrhage near the vitreous base, where the surface tension counteracts the aspiration force during vitrector cutting intervals, stabilizing the retinal surface and facilitating safe completion of PVD (Additional file 2). The combination of high surface tension with high cut rates and low vacuum settings, along with modern small-gauge port designs positioned closer to the tip, creates a dynamic stabilization effect that is especially advantageous in patients with PDR who are at risk of active intraoperative bleeding.

**Fig. 4:**
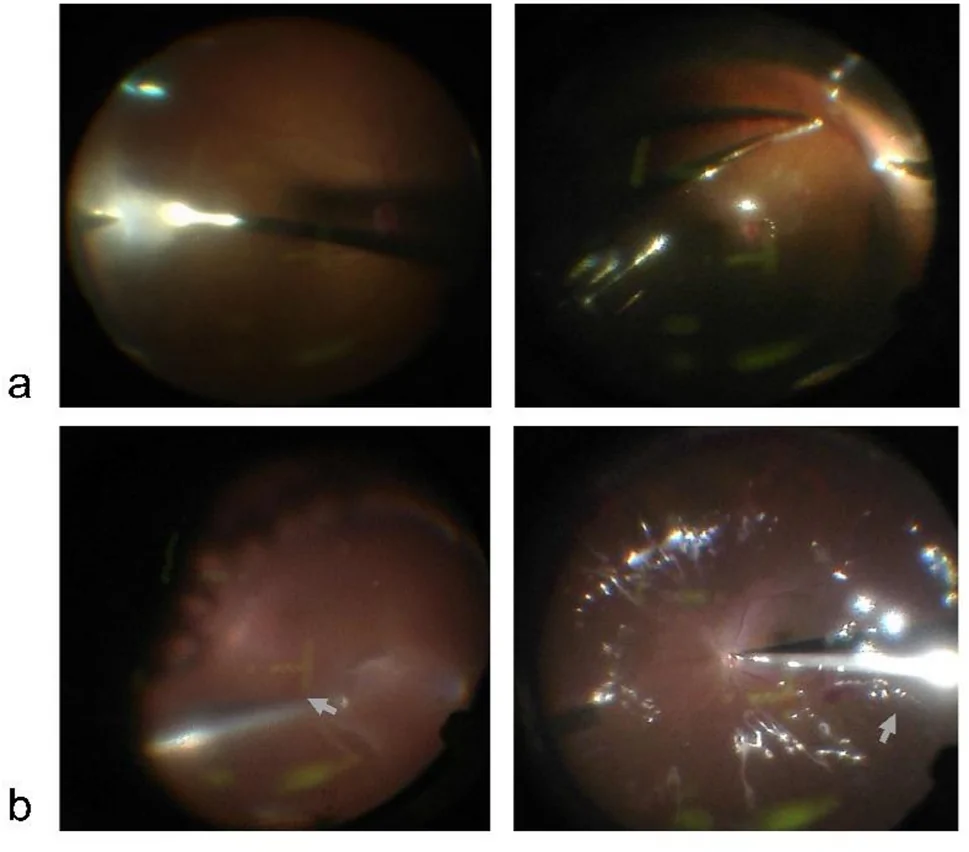
Comparing vitrectomy under air and fluid infusion. **(a) (Left)** Direct peripheral vitrectomy under air infusion. **(Right)** Fluid infusion provides a limited visual field, necessitating dynamic scleral depression for adequate removal of the peripheral vitreous. **(b)** Visualization of the same retinal bleeding point under air (left) versus under fluid (right) in a case of vitreous hemorrhage due to BRVO

Third, due to the significant refractive index difference in an air environment [16], surgeons often achieve a wider retinal field of view, allowing clear observation of residual hemorrhagic vitreous at the retinal periphery without requiring external pressure. This facilitates easier excision of peripheral vitreous (Fig. 4a). It is well established that vitreous hemorrhage is frequently associated with retinal pathologies, such as peripheral retinal breaks and retinal neovascularization. Under conventional BSS infusion, these peripheral lesions are often obscured by hemorrhagic opacities, rendering them difficult to detect. The wide-angle viewing advantage provided by vitrectomy under air enables surgeons to conduct more comprehensive examinations of the peripheral retina, facilitating timely identification and management of underlying pathologies. Moreover, during complete retinal examination under air, even minute bleeding points or neovascular lesions can be clearly identified, which is of paramount importance for achieving precise intraoperative hemostasis and preventing postoperative rebleeding (Fig. 4b).

The comparable postoperative BCVA and CMT between groups at 1 month suggest that the air technique does not compromise short-term visual or anatomical recovery despite the different intraoperative medium. Both infusion media are capable of achieving posterior vitreous detachment; rather than producing a more complete PVD, the clearer air–vitreous interface may primarily allow the surgeon to confirm more readily whether PVD and posterior cortical vitreous removal are complete. Although postoperative complications were numerically fewer in the air group (1 vs. 3 eyes), the sample size was insufficient for a meaningful statistical comparison of complication rates, and we therefore refrain from drawing conclusions about relative safety. We can only hypothesize that more thorough posterior hyaloid removal under improved visualization, together with the shorter effective vitrectomy time, might contribute to a lower risk of postoperative PVR and retinal re-detachment; this remains speculative and requires confirmation in larger studies.

Vitrectomy under air also has several limitations and potential risks that warrant consideration. In phakic eyes, prolonged air infusion may cause transient posterior capsule opacification; whether combined phacovitrectomy with air infusion is feasible was not evaluated in this study, because such cases were excluded, and this requires further study. In our clinical observations, condensation on the posterior surface of the intraocular lens occasionally occurred after fluid–air exchange in pseudophakic patients with posterior capsule defects; this fogging could be eliminated by coating with viscoelastic material to maintain clear visualization. Several technique-specific risks should also be emphasized. In eyes with unrecognized vitreoretinal traction or peripheral retinal breaks, air infusion may exert traction on the retina and could allow air to pass into the subretinal space; the technique is therefore contraindicated in eyes with pre-existing tractional retinal detachment, in which instrument entry into tissue planes may damage normal tissue. In addition, peripheral vitrectomy under air can be technically more demanding than under BSS because of the surface tension of air and altered instrument dynamics, so the technique likely entails a learning curve and is dependent on surgeon experience. These considerations underscore the importance of careful patient selection and adequate surgical experience when adopting this approach [21].

Several limitations of this study merit consideration. First, this was a non-randomized, retrospective study with a relatively small sample size of 16 eyes per group, which limits statistical power, particularly for the comparison of complication rates; because allocation was not randomized, selection bias cannot be excluded. Second, there was a significant imbalance in disease etiology between groups (PDR 87.5% in the air group vs. 37.5% in the BSS group; all four RRD eyes in the BSS group; *p* = 0.007). Although RRD procedures are often longer and more complex than vitrectomy for vitreous hemorrhage of other causes, in the present series the RRD eyes did not in fact have longer effective vitrectomy times, and excluding them slightly increased rather than reduced the between-group difference. To address this confounding, we performed a subgroup analysis excluding RRD eyes and a multivariable regression adjusting for etiology, both of which showed that air infusion remained associated with a shorter effective vitrectomy time; nonetheless, residual confounding cannot be fully excluded. Third, effective vitrectomy time was timed in real time by a single, non-masked surgical assistant using a stopwatch rather than by masked review of recorded video, so measurement imprecision and potential observer bias cannot be excluded, and inter-rater reliability was not assessed. Total surgical time was not analysed; because it is largely determined by adjunctive steps performed under BSS in both groups (ILM peeling, endolaser, membrane removal, and tamponade) that are independent of the infusion medium, effective vitrectomy time was considered the more specific measure of the efficiency difference attributable to the infusion medium. Fourth, the 1-month follow-up is too short to assess longer-term endpoints such as recurrent vitreous hemorrhage, PVR, retinal re-detachment, and stabilization of visual acuity, so no conclusions about long-term safety or efficacy can be drawn. Fifth, all surgeries were performed by a single experienced surgeon; because vitrectomy under air may involve a learning curve and altered intraoperative ergonomics, the reproducibility and generalizability of these findings to other surgeons and centers require confirmation. Future prospective, randomized, multicenter studies with larger samples and longer follow-up are warranted to validate our findings.

## Conclusions

In this retrospective comparison of BSS infusion versus air infusion for the removal of dense vitreous hemorrhage, the two techniques achieved comparable short-term visual and anatomical outcomes, while air infusion was associated with a shorter effective vitrectomy time. By exploiting the physical properties of air, this approach may improve intraoperative visualization of the hemorrhagic vitreous and could represent a useful alternative in selected cases. Given the retrospective design, small sample, etiologic imbalance, and short follow-up, these findings should be regarded as preliminary; larger prospective studies with longer follow-up are warranted to confirm them.

## Supplementary Information

Below is the link to the electronic supplementary material.

Supplementary Material 1: Additional file 1: Video S1. Surgical procedure of vitrectomy under air infusion demonstrating the key steps. (MP4)

Supplementary Material 2: Additional file 2: Video S2. Demonstration of posterior vitreous detachment completion under air infusion showing surface tension effects. (MP4)

## Data Availability

The datasets used and analyzed during the current study are available from the corresponding author upon reasonable request.

## Abbreviations

VH: Vitreous hemorrhage
PPV: Pars plana vitrectomy
BSS: Balanced salt solution
PDR: Proliferative diabetic retinopathy
RVO: Retinal vein occlusion
RRD: Rhegmatogenous retinal detachment
VEGF: Vascular endothelial growth factor
PVD: Posterior vitreous detachment
PVR: Proliferative vitreoretinopathy
BCVA: Best-corrected visual acuity
CMT: Central macular thickness
OCT: Optical coherence tomography
IOP: Intraocular pressure
AL: Axial length
ILM: Internal limiting membrane
PRP: Panretinal photocoagulation
logMAR: Logarithm of the minimum angle of resolution
STROBE: Strengthening the Reporting of Observational Studies in Epidemiology
SD: Standard deviation
SE: Standard error
CI: Confidence interval
BRVO: Branch retinal vein occlusion
